# Differentiation Defect Into GABAergic Neurons in Cerebral Organoids From Autism Patients

**DOI:** 10.1111/cns.70449

**Published:** 2025-06-02

**Authors:** Sai Hali, Xuerui Yao, Guo Hao, Zhe‐Long Jin, Kun Fu, Yunxiao Li, Lin Wang, Heejeong Yoo, Hyeonwoo La, Chanhyeok Park, Kwonho Hong, Chan Young Shin, Dong‐Hun Woo, Choongseong Han, Xiong Jin, Shifeng Zhu, Wenquan Zou, Nam‐Hyung Kim, Kee‐Pyo Kim, Leshuai W. Zhang, Dong Wook Han

**Affiliations:** ^1^ Department of Advanced Translational Medicine, School of Medicine Konkuk University Seoul Republic of Korea; ^2^ NUOXINTE Biotechnology Suzhou China; ^3^ Institute of Neurology, Jiangxi Academy of Clinical Medical Sciences, Department of Neurology, the First Affiliated Hospital, Jiangxi Medical College Nanchang University Nanchang China; ^4^ Guangdong Provincial Key Laboratory of Large Animal Models for Biomedicine, School of Biotechnology and Health Sciences Wuyi University Jiangmen China; ^5^ Healthcare International Innovation Institute Jiangmen China; ^6^ Guangdong University of Technology Guangzhou China; ^7^ Research and Development Qingdao Haier Biotech co. Ltd Qingdao China; ^8^ Department of Psychiatry Seoul National University College of Medicine Seoul Republic of Korea; ^9^ Department of Psychiatry Seoul National University Bundang Hospital Seongnam Gyeonggi Republic of Korea; ^10^ Department of Stem Cell and Regenerative Biotechnology, the Institute of Advanced Regenerative Science Konkuk University Seoul Republic of Korea; ^11^ Department of Pharmacology and Department of Advanced Translational Medicine, School of Medicine Konkuk University Seoul Republic of Korea; ^12^ Department of Stem Cell Biology NEXEL co., Ltd Seoul Republic of Korea; ^13^ Department of Life Sciences College of Medicine, the Catholic University of Korea Seoul Republic of Korea; ^14^ School of Life Sciences Suzhou Medical College of Soochow University Suzhou China; ^15^ State Key Laboratory of Radiation Medicine and Protection, Collaborative Innovation Center of Radiation Medicine of Jiangsu Higher Education Institutions Soochow University Suzhou China

**Keywords:** autism spectrum disorder, cerebral organoids, disease modeling, drug screening

## Abstract

**Objectives:**

Autism spectrum disorder (ASD) is a neurodevelopmental condition that affects social communication and behaviors. While previous studies using animal models have substantially expanded our knowledge about ASD, the lack of an appropriate human model system that accurately recapitulates the human‐specific pathophysiology of ASD hinders the precise understanding of its etiology and the development of effective therapies. This study aims to replicate pathological phenotypes in cerebral organoids derived from idiopathic ASD patients and to conduct proof‐of‐concept research for the development of ASD therapeutics.

**Methods:**

We conducted an in vitro disease modeling study using cerebral organoids derived from three idiopathic ASD patients. Additionally, we performed organoid‐based phenotypic drug screening to identify potential therapeutic compounds that could ameliorate the phenotypes observed in cerebral organoids derived from idiopathic ASD patients.

**Results:**

Here we show that cerebral organoids derived from idiopathic ASD patients display malformation of the ventricular zones and impaired early neuronal differentiation. Through organoid‐based phenotypic drug screening, we successfully generated cerebral organoids with normal tissue architecture in which the delayed neuronal differentiation could also be accelerated. Notably, cerebral organoids from ASD patients exhibited a reduced number of GABAergic neurons compared to healthy controls, resulting in an imbalance in the excitatory and inhibitory neuron ratio. The differentiation defects into GABAergic neurons in patient‐derived cerebral organoids could be rescued by treating with either IGF1 or Gabapentin, a GABA agonist.

**Conclusions:**

Our findings provide a framework for utilizing patient‐derived cerebral organoids in the development of personalized pharmaceutical treatment for ASD.

## Graphical abstract

Summary of in vitro disease modeling and drug screening using ASD patient‐derived COs. This figure highlights the major phenotypes observed in CO^ASD^ and the therapeutic effects of each compound screened in this study.

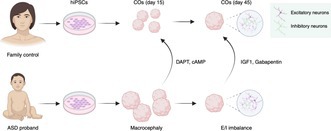

## 1 Introduction

Autism spectrum disorder (ASD) is a group of neurodevelopmental disorders characterized by persistent deficits in social communication, restricted interests, and repetitive behavioral patterns [1]. Approximately 15%–20% of individuals with ASD have been associated with environmental or genetic factors, including single‐gene disorders, copy number variations, and chromosomal abnormalities [2]. However, the vast majority of cases remain idiopathic, lacking a clear identifiable cause. The increasing prevalence rates of ASD underscore the need to identify reliable biomarkers and therapeutic targets.

Animal models have been extensively used to investigate the underlying disease mechanisms of ASD. Although previous approaches based on animal models have substantially advanced our understanding of ASD, they have limitations in replicating the human‐specific neurodevelopmental processes and tissue architecture, resulting in an incomplete recapitulation of unique human‐specific ASD phenotypes [3, 4, 5]. Thus, there is an urgent need for the development of human‐based in vitro model systems. The utilization of patient‐derived human induced pluripotent stem cell (hiPSC) technology has introduced a novel concept of patient‐specific disease modeling and drug screening [6, 7]. Conventional two‐dimensional (2D) differentiation technology typically enables the production of highly uniform, singular cell types. The robustness of 2D cell culture, such as scalability and simplicity, makes hiPSC‐derived 2D neurons particularly suitable for both basic research and high‐throughput screening. However, hiPSC‐derived 2D cell types often lack key features necessary to accurately replicate the complexity of human brain tissues, such as cellular diversity and essential cell‐to‐cell and cell‐to‐matrix interactions. Indeed, previous reports using neurons differentiated from ASD patient‐derived hiPSCs [8, 9] described abnormal phenotypes on cellular levels, such as aberrancies in soma sizes [10], neurite lengths [11], synaptic structures [12], and electrophysiological properties [13]. However, it should be acknowledged that previous in vitro ASD modeling studies based on 2D differentiation protocols could not fully recapitulate in vivo disease pathology observed in patient brains [14, 15] including altered brain sizes, altered distribution of neuronal subtypes, and imbalance between excitatory and inhibitory neurons.

Using the self‐organizing property of human pluripotent stem cells (hPSCs), previous studies have demonstrated the generation of miniaturized three‐dimensional (3D) organoids that share similar tissue architectures with their in vivo counterparts [16, 17]. Recent advances also described the generation of region‐specific brain organoids representing various brain regions, such as cerebral cortex [18], forebrain [19, 20], midbrain [21, 22], optic cups [23], hippocampus [24], pituitary gland [25], thalamus [26], hypothalamus [27], choroid plexus [28], cerebellum [29], brainstem [30], and spinal cord [31] from human PSCs. Brain organoids contain multiple cell types [22] with tissue architectures similar to the developing human brain [17], making them valuable tools for disease modeling and screening potential therapeutics for neurodevelopmental disorders like ASD. Indeed, previous studies demonstrated that cerebral organoids (COs) from idiopathic or monogenic syndromic ASD patients could phenocopy key pathological features of ASD patients, including altered electrophysiological properties, macrocephaly, and an imbalance between excitatory and inhibitory neurons (E/I imbalance) [32, 33, 34, 35, 36, 37, 38, 39, 40, 41]. While previous studies have shown promising results in reversing altered neuronal activity observed in patient‐derived COs using drugs, further efforts for the development of potential drugs capable of effectively modifying the altered tissue architectures in patient‐derived COs, such as macrocephaly and the altered distribution of glutamatergic and/or GABAergic neurons leading to E/I imbalance, are essentially required. We previously described the therapeutic potential of Retigabine, an anti‐epileptic and anti‐convulsant drug, for the inhibitory neuron‐specific defect that might contribute to E/I imbalance using mouse COs from contactin‐associated protein‐like 2 (Cntnap2) knockout mice [42]. However, achieving a similar pharmaceutical approach leading to the phenotypic rescue of histopathological symptoms, such as macrocephaly and altered distribution of glutamatergic and/or GABAergic neurons in patient‐derived COs has yet to be accomplished.

In the current study, we successfully recapitulated major ASD phenotypes, including macrocephaly‐like phenotype, altered neuronal differentiation pattern, and reduced differentiation into GABAergic neurons in COs from idiopathic ASD patients. Furthermore, we conducted screenings of small molecules or drugs that efficiently ameliorate all the aforementioned ASD‐related phenotypes observed in patient‐derived COs. Our findings suggest that patient‐derived COs hold significant promise as a valuable platform for developing personalized pharmaceutical interventions for ASD treatment.

## 2 Materials and Methods

### 2.1 iPSC Lines Used in This Study

Six hiPSC lines were used in this study: three lines from healthy family controls and three from ASD probands. The primary hiPSC lines (CO^Control#1^ and CO^ASD#1^) that were mainly used for this study were obtained from the Coriell Institute for Medical Research. Additional hiPSC lines (CO^Control#2^, CO^Control#3^, CO^ASD#2^, and CO^ASD#3^) from two other idiopathic patients and their corresponding healthy family controls were generated from freshly isolated PBMCs as we described previously [43]. Informed consent was obtained from each individual before generating the hiPSC lines, and ethical approval was obtained from Seoul National University Bundang Hospital.

### 2.2 iPSC Generation

Lentiviruses encoding reprogramming factors were produced by transfecting HEK293 cells (ATCC) with 3 μg of pUMVC (#8449, Addgene), 1.5 μg of pCMV‐VSV‐G (#8485, Addgene), and 4.5 μg of pRRL‐OSKM using 27 μl of Fugene 6 transfection reagent (Promega) in 600 μl of Opti‐MEM (Invitrogen). The lentivirus‐containing supernatants were collected, filtered, concentrated, and stored at −80°C before use.

After two rounds of transduction with lentiviruses containing reprogramming factors in the presence of 8 μg/ml protamine sulfate (Sigma), the cells were plated on a 6‐well plate precoated with CF1 feeder cells. After 3 weeks, hiPSC colonies were manually picked, replated on a Matrigel‐coated plate, and cultured in mTeSR (STEMCELL Technologies). hiPSCs were cultured at 37°C, 90% humidity, and 5% CO2 with a daily medium change.

### 2.3 Cell Culture

hPSCs, including hESCs and hiPSCs, were cultured on hESC‐qualified Matrigel (Corning) coated 6‐well plates using mTeSR1 (STEMCELL Technologies). All hPSC lines were maintained in a 5% CO2 incubator at 37°C and routinely tested for contamination, confirming them as mycoplasma‐free. For passaging hPSCs, ReLeSR (STEMCELL Technologies) was used according to the manufacturer's instructions.

### 2.4 Generation of COs From hPSCs

The hPSCs were dissociated into single cells using TrypLETM (Gibco) to generate COs from hPSCs. A total of 9 × 10 [3] cells were plated per well in ultra‐low attachment 96‐well round‐bottom plates (Corning) for the generation of EBs using EB forming medium (EBM). The EBM consisted of DMEM/F12 (Corning) supplemented with 20% KSR (Gibco), 1% penicillin/streptomycin (P/S) (Gibco), GlutaMAXTM (Gibco), 1% NEAA (Gibco), 55 μM ß‐mercaptoethanol (Gibco), 1 μg/ml of heparin (Sigma), 3% fetal bovine serum (FBS) (Seradigm), 4 ng/ml of bFGF (Peprotech), and 50 μM Y‐27632 (Calbiochem). At 24 h after plating, the medium was changed to brain organoid generation medium (BGM), containing a 1:1 mix of DMEM/F12 (Corning) and Neurobasal Medium (Gibco) supplemented with 100x N2 supplement (Gibco), 50x B27 without vitamin A (Gibco), 1% penicillin/streptomycin (P/S) (Gibco), 1% GlutaMAXTM (Gibco), 1% NEAA (Gibco), 55 μM ß‐mercaptoethanol (Gibco), and 1 μg/ml of heparin (Sigma). BGM was replaced every other day for up to 15 days. For early neuroectodermal commitment and telecephalon specification (days 1 to 7), 2 μM dorsomorphin (Sigma) and 2 μM A83‐01 (Peprotech) were used. The specification into the ventral telecephalon (days 4 to 15) was induced by treating EBs with 100 nM of SAG (Peprotech) from day 4 to 15 [44, 45, 46, 47]. On day 7, COs were embedded into growth factor‐reduced Matrigel (Corning) droplets in ultra‐low‐attachment 6‐well plates (Corning). Embedded COs were cultured in stationary conditions for 4 days, then transferred to an orbital shaker (Stuart) with brain organoid maturation medium (BMM). BMM consisted of a 1:1 mix of DMEM/F12 (Corning) and Neurobasal Medium (Gibco) supplemented with 100x N2 supplement (Gibco), 50x B27 (Gibco), 1% penicillin/streptomycin (P/S) (Gibco), 1% GlutaMAX (Gibco), 1% NEAA (Gibco), 55 μM ß‐mercaptoethanol (Gibco), 1 μg/ml of heparin (Sigma), 10 ng/ml of BDNF (Peprotech), 10 ng/ml of GDNF (Peprotech), 200 μM ascorbic acid (Peprotech), and 125 μM cAMP (Peprotech). BMM was replaced every other day for up to 100 days.

### 2.5 Drug Treatment

Small molecules such as DAPT (10 μM, Tocris), SU5402 (5 μM, Biovision), PD0325901 (1 μM, Tocris), and cAMP (50 μM, Sigma) were administered three times on days 7, 9, and 11, with analysis performed on day 15. All drugs, including Gabapentin (100 μM, Sigma), Ganaxolone (50 ng/ml, Tocris), Vigabatrin (60 μM, Sigma), Tiagabine hydrochloride (50 ng/ml, Tocris), Retigabine (10 μM, Tocris), and IGF‐1 (20 ng/ml, Peprotech), were administered for 10 days (from day 35 to 45), with analysis conducted on day 45.

### 2.6 Cryosection

The COs were fixed with 4% paraformaldehyde (Sigma) for 15 to 20 min at room temperature. They were then washed three times with PBS, transferred to 30% sucrose solution, and kept at 4°C until COs sank to the bottom. Subsequently, COs were transferred into embedding medium (Tissue‐Tek OCT Compound 4583, Sakura Finetek), snap‐frozen on dry ice, and stored at −80°C. For immunohistochemistry, 16 μm‐thick sections were obtained using a cryostat (Leica).

### 2.7 Immunohistochemistry

The dried cryosections were washed with PBS to remove excess OCT and blocked in 10% FBS (Seradigm), 0.5% Triton X‐100 diluted in PBS for 1 h at room temperature. The sections were then incubated overnight at 4°C with the appropriate primary antibodies diluted in a solution containing 10% FBS and 0.5% Triton X‐100. The primary antibodies were washed clearly with PBS, and then the sections were incubated with the appropriate secondary antibodies containing 10% FBS and 0.5% Triton X‐100 for 1 h. DAPI was used for nuclear staining. Primary antibodies used for immunohistochemistry were as follows: SOX2 (R&D system), TUJ1 (Biolegend), PAX6 (Abcam), FOXG1 (Abcam), Ki67 (BD Bioscience), MAP2 (Merk Millipore), SYN1 (Cell Signaling Technology), SYP (Cusabio), PSD95 (Thermo Fisher), GLUT (Cusabio), GABA (Sigma), GFAP (CiteAb), MBP (Abcam), LAMININ (Abcam), REELIN (Merk Millipore), TBR1 (Abcam), TBR2 (Abcam), ZO‐1 (Thermo Fisher), CTIP2 (Abcam), SATB2 (Abcam), Caspase 3 (Cell Signaling), and vGlut1 (Abcam). Cryosections were mounted for microscopy on glass coverslips and imaged on a Zeiss (LSM 710 META) and Leica SP8 with a 10×, 20×, 40×, or 63× objective. Images were processed using Zen software (version 8.0, Zeiss).

### 2.8 Gene Expression Analysis

Total RNA was isolated using the Hybrid‐RTM RNA isolation kit (GeneAll). cDNA was synthesized with the High‐Capacity cDNA Reverse Transcription Kit (Applied Biosystems). Quantitative RT‐PCR (qPCR) was carried out with the SYBR Green PCR Master Mix (Applied Biosystems) using the ABI 7500 real‐time PCR system (Applied Biosystems). Relative expression levels were calculated by using ^−ΔΔ^the 2^Ct method.

### 2.9 RNA‐Seq Library Construction

Polyadenylated RNA was purified using oligo‐dT magnetic beads, fragmented, and reverse transcribed into single‐stranded cDNA using random hexamer primers and reverse transcriptase. Actinomycin D was added to inhibit DNA‐dependent second‐strand synthesis. Double‐stranded cDNA was generated by removing the RNA template and synthesizing the second strand in the presence of dUTP. Library preparation was performed using the TruSeq Stranded mRNA Library Prep Kit (Illumina, 20020595) following the manufacturer's instructions. The final library was sequenced on an Illumina NovaSeq 6000 sequencer.

### 2.10 Post‐Sequencing Analysis

After sequencing, paired‐end reads were mapped to the hg38 UCSC human genome using STAR (v2.4.2a) [48]. Read quantification and normalization to FPKM were performed using Cuffquant and Cuffnorm from the Cufflinks package (v2.2.1) [49]. Differentially expressed genes (DEGs) were identified in R (v4.3.0) [50], defined as genes with an FPKM value > 2 and a fold change > 2 between two samples. Gene ontology analysis of DEGs was conducted using DAVID (v2021 update) [51]. Heatmaps of gene expression levels were generated using the heatmap2 function from the ggplot2 (v3.5.1) [52] package in R.

### 2.11 Bisulfite Sequencing

Bisulfite conversion was performed using an EZ DNA methylation kit (Zymo Research) according to the manufacturer's instructions. PCR was carried out using HotStarTaq DNA Polymerase, and the resulting PCR products were cloned into pCRII TOPO vector (Invitrogen). Individual clones were subjected to sequencing. Sequencing data were analyzed using QUMA software (http://quma.cdb.riken.jp). The primer sequences used for PCR are as follows: sense ATTTGTTTTTTGGGTAGTTAAAGGT, antisense CCAACTATCTTCATCTTAATAACATCC.

### 2.12 Karyotyping

The hPSCs (passage 10) were treated with 10 μg/mL colcemid (Gibco) and incubated with hypotonic solution for 25 min at 37°C. After dissociation and centrifugation, cells were resuspended in fixative solution (methanol: acetic acid = 3: 1). The resuspended cells were transferred onto cold, wet slides, and the slides were then subjected to GTG‐banding.

### 2.13 Teratoma Assay

5 × 10^6^ single‐cell dissociated hPSCs (passage 10), resuspended in 100 μL of ice‐cold 1:1 mixture of hESC medium and Matrigel (BD Biosciences), were injected into five‐week‐old immunodeficient NOD‐SCID mice according to the approved institutional animal protocol.

About 9 weeks after injection, teratomas were collected surgically, fixed in 4% formaldehyde, and embedded in paraffin. Teratomas were sectioned using the microtome (Nikon) and stained with hematoxylin and eosin (Sigma).

### 2.14 Statistical Analysis

For statistical analysis, t‐tests were applied to compare two groups. ANOVA analyses were used for comparisons of data with more than two groups. All the values were calculated from at least triplicate experiments, and the *p* values were presented as *P < 0.05; **P < 0.01; ***P < 0.001.

## 3 Results

### 3.1 Generation of Cerebral Organoids From hPSCs

**FIGURE 1:**
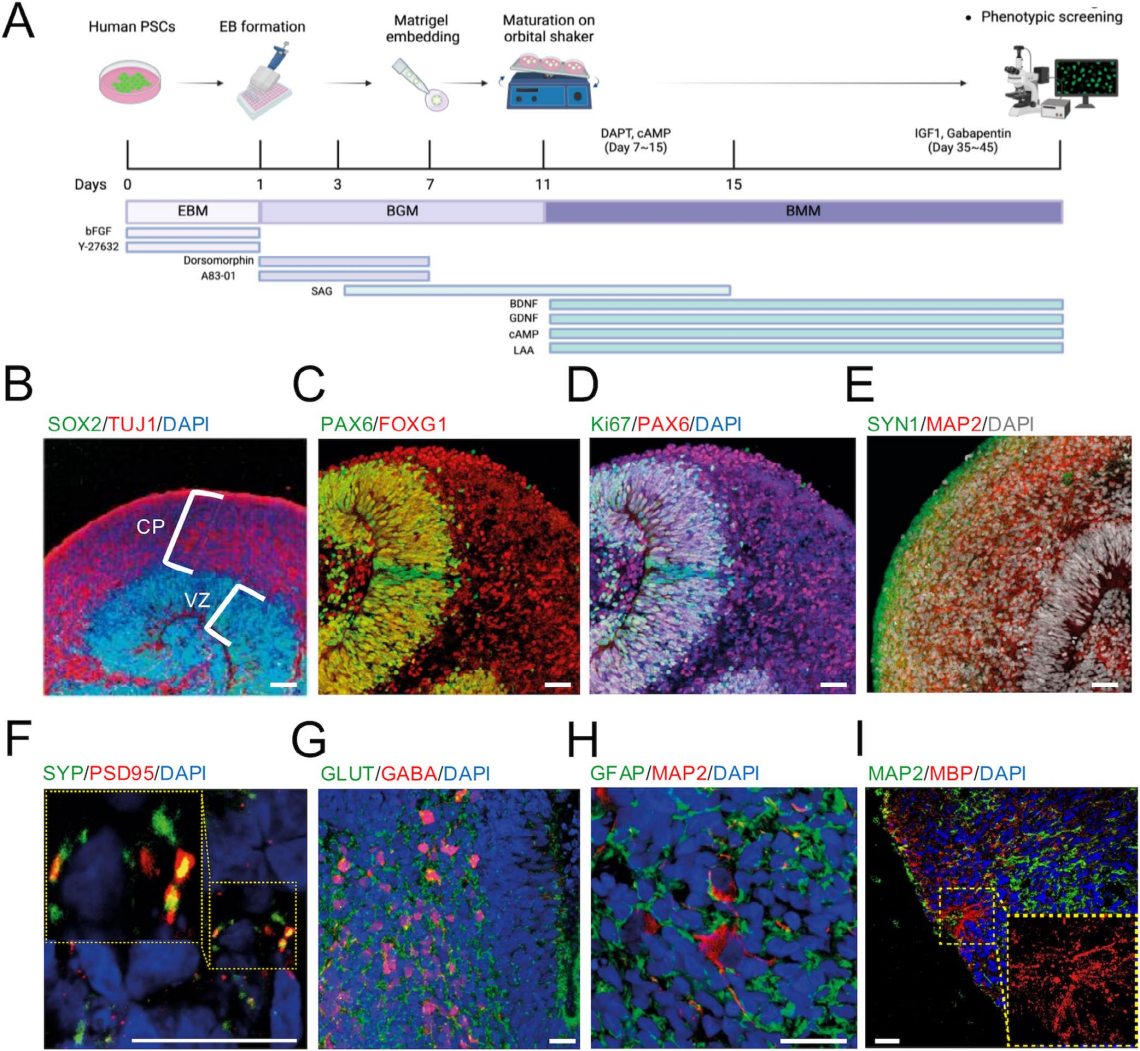
Generation of COs from hPSCs. (A) Schematic illustration of the procedure for generating COs. The procedures and timelines for generating COs are described. (B, C, D, E) Confocal images of COs showing expression patterns of SOX2/TUJ1 (B), PAX6/FOXG1 (C), Ki67/PAX6 (D), and SYN1/MAP2 (E). (F) Confocal image showing the co‐localization of SYP and PSD95 in COs. (G, H, I) Confocal images of COs showing expression patterns of GLUT/GABA (G), GFAP/MAP2 (H), and MBP/MAP2 (I). Scale bars represent 100 μm. COs, Cerebral organoids.

The generation of COs from hPSCs can be achieved through a step‐wise protocol, which involves neuroectoderm specification, basal‐apical lamination, and maturation (Figure 1A). To initiate the generation of COs from human embryonic stem cells (hESCs), we first plated 9x10 [3] single‐cell dissociated hESCs on u‐bottom 96‐well plates for forming embryoid bodies (EBs). To induce early neuroectodermal commitment of hESCs, we treated dual SMAD inhibitors (2 μM dorsomorphin + 2 μM A83‐01) on EBs as in our previous studies [22, 53]. After inducing basal‐apical lamination by embedding COs into Matrigel droplets, COs were cultured on an orbital shaker for maturation (Figure 1A).

The generated COs at the early stage (day 22) exhibited several key features reminiscent of the developing human fetal brain [54] (Figure S1A,B). These included a SOX2^+^ ventricular zone (VZ), a single layer of TBR1^+^/TUJ1^+^ cortical plate, a Laminin^+^ basement membrane, and Reelin^+^ Cajal–Retzius neurons. At day 35, we observed the formation of the outer subventricular zone (oSVZ) and inner subventricular zone (iSVZ), accompanied by cortical plates comprising distinct neuronal layers (CTIP2^+^ deep layer and SATB2^+^ superficial layer) (Figure S1C,D). By day 45, the COs exhibited a well‐defined stratified tissue architecture containing a SOX2^+^/PAX6^+^/Ki67^+^ VZ containing proliferating neural progenitor cells (Figure 1B–D), a further thickened cortical plate (Figure 1E), and a Reelin^+^/Laminin^+^ marginal zone (Figure S1C), mirroring the developmental status of the human cerebral cortex during the second trimester of gestation (Figure S1B). We were also able to find the co‐localization of a presynaptic marker, Synaptophysin (SYP), and a postsynaptic marker PSD95 in COs at day 45 (Figure 1F), indicating the presence of synaptic connections. Furthermore, the COs displayed diverse cellular components, such as distinct neuronal subtypes, such as GABAergic and glutamatergic neurons (day 45) (Figure 1G), as well as glial cells, including astrocytes and oligodendrocytes (day 60) (Figure 1H,I). Taken together, our data indicate that COs derived from hESCs exhibit remarkable structural similarities to the developing human cerebral cortex during the second trimester of gestation.

### 3.2 Malformation of Ventricular Zones With Delayed Neural Differentiation in ASD Organoids

**FIGURE 2:**
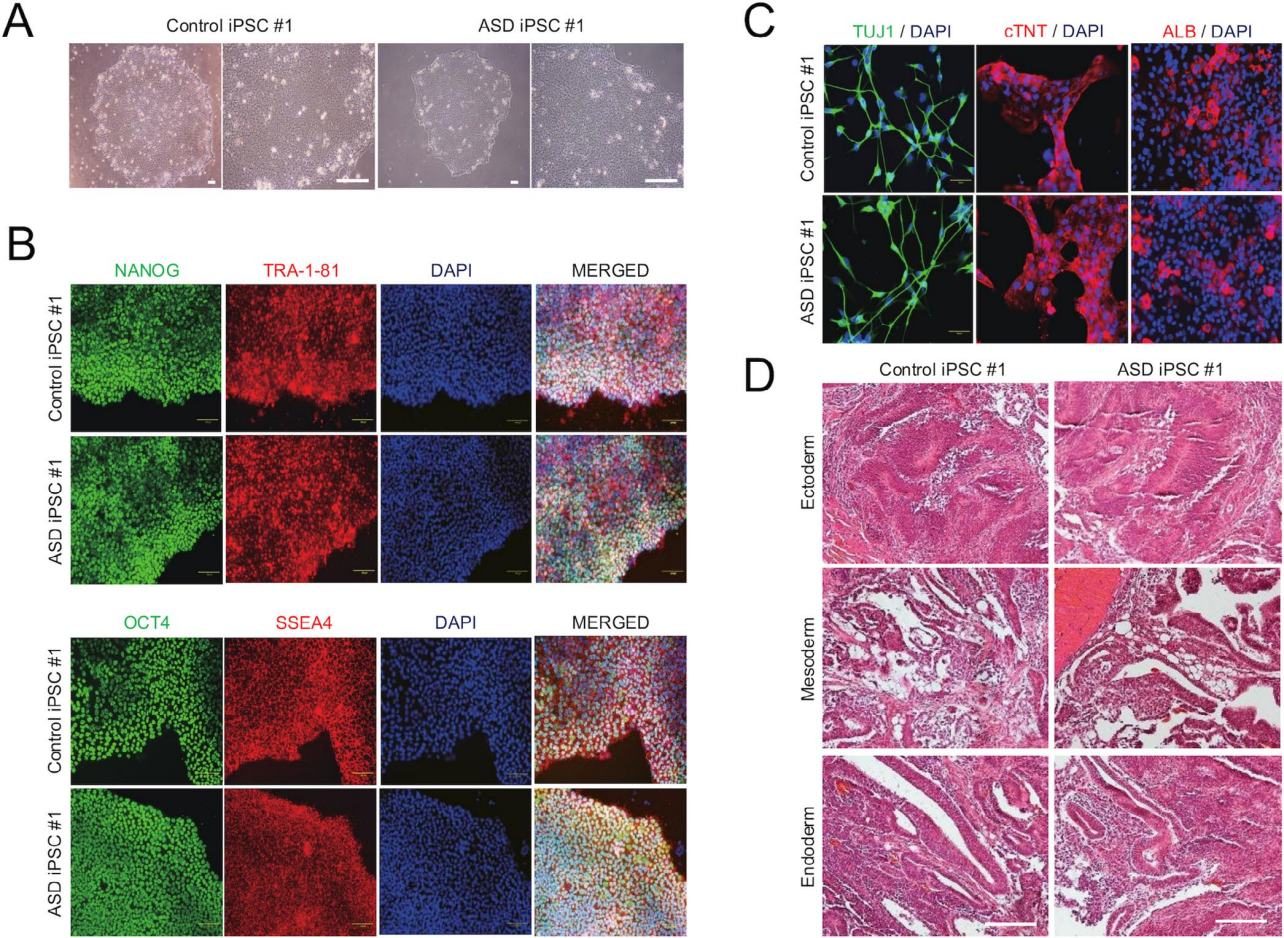
Pluripotency of hiPSC lines from idiopathic ASD patients and family controls. (A) Morphology of hiPSC lines. (B) Expression of pluripotency markers in hiPSC lines. (C, D) In vitro (C) and in vivo (D) differentiation potentials of hiPSC lines into three germ layers. Scale bars represent 100 μm.

For the in vitro modeling of idiopathic ASD, we obtained hiPSC lines from an idiopathic ASD proband and its family control from the Coriell Institute for Medical Research. Additionally, we generated hiPSC lines from two other idiopathic patients and their corresponding healthy family controls [43]. As in our previous study [43], all the hiPSC lines exhibited similar morphology (Figure 2A), gene expression patterns (Figure 2B and S2A), both in vitro and in vivo differentiation potentials (Figure 2C,D), epigenetic status (Figure S2B), and normal karyotypes (Figure S2C) compared to hESCs, indicating that hiPSC lines derived from both idiopathic ASD patients and healthy family controls retain pluripotential characteristics.

**FIGURE 3:**
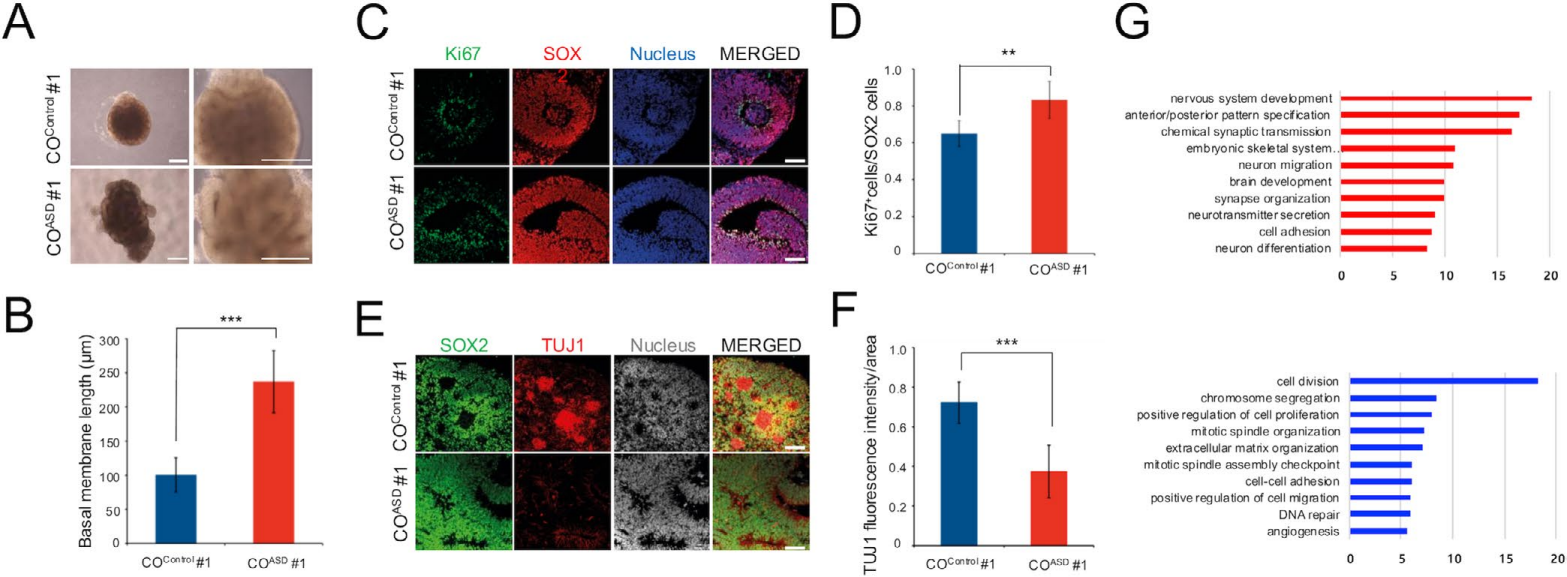
Malformation of ventricular zones in ASD organoids. (A) Representative bright field images of CO^control^ and CO^ASD^ at day 15. (B) Average basal membrane lengths of CO^control^ and CO^ASD^ at day 15 (*p* = 3.36 x 10^−6^). (C, D) Confocal images (C) and average numbers (D) of Ki67+ proliferating progenitor cells in VZ of CO^control^ and CO^ASD^ at day 15 (*p* = 3.77 x 10^−3^). (E, F) Confocal images (E) and average numbers (F) of TUJ1+ neurons in CO^control^ and CO^ASD^ at day 15 (*p* = 1.5 x 10^−4^). (G) Gene ontological analysis of differentially expressed genes between CO^control^ and CO^ASD^ at day 15. A Total of 15 COs Per Group Were Subjected to RNAseq Analysis. Scale bars represent 100 μm. Data are presented as mean ± SD from three independent experiments. CO^ASD^, COs from ASD hiPSC lines; CO^Control^, COs from control hiPSC lines.

Using our optimized protocol (Figure 1A), we next generated COs from both control and ASD hiPSC lines (hereafter referred to as CO^control^ and CO^ASD^, respectively). The efficiency of EB formation and early CO generation was comparable between the control‐ and patient‐derived hiPSC lines (data not shown). However, compared with CO^control^, CO^ASD^ displayed enhanced sizes and abnormally elongated shapes of VZ areas (Figure 3A,B) that could be explained by an increased number of Ki67^+^ proliferating progenitor cells in the VZ of CO^ASD^ compared to that in CO^control^ (Figure 3C,D). This increased number of progenitor population in CO^ASD^ strongly prompted us to investigate the early neuronal differentiation process in CO^ASD^. Thus, we next investigated the early neuronal differentiation in COs (day 15) by checking the number of neurons expressing TUJ1. In CO^control^, TUJ1^+^ neurons were readily observed (Figure 3E). In contrast, TUJ1^+^ cells were barely observed in CO^ASD^, even after being cultured for the same duration as CO^control^ (Figure 3E,F). Similar phenotypes, including enlarged VZ areas, increased numbers of proliferating progenitors, and reduced numbers of early differentiating neurons, were also observed in CO^ASD^ derived from two other patients, albeit with varying degrees (Figure S3A,B). RNA‐sequencing (RNAseq) analysis further confirmed this neural differentiation defect in CO^ASD^. Genes highly enriched in CO^Control^ were associated with neuronal processes such as neuron migration, axon guidance, nervous system development, and neuron differentiation (Figure 3G). In contrast, only non‐neuronal processes were prominently enriched in CO^ASD^ (Figure 3G). Similarly, CO^Control^ exhibited higher expression levels of both GABAergic and glutamatergic progenitor markers compared to CO^ASD^ (Figure S3C), further supporting the impairment in early neuronal differentiation in CO^ASD^. Collectively, our data indicate that COs derived from idiopathic ASD patients exhibit malformations of VZ, characterized by enlarged rosette areas and impaired early neural differentiation, contributing to a macrocephaly‐like phenotype.

### 3.3 Early Pharmaceutical Intervention Facilitates the Rescue of Macrocephaly Phenotype in ASD Organoids

The manifestation of macrocephaly typically appears during the perinatal or early postnatal stages and persists throughout later stages of life. Several previous studies have described the macrocephaly phenotype in COs derived from either ASD patient‐derived hiPSCs or loss‐of‐function PSC models using genome editing technology [32, 33, 34, 35]. However, it is worth noting that the macrocephaly phenotypes observed in COs from these previous studies are commonly transient, and thus, only the early stage of COs derived from ASD patients exhibited the macrocephaly phenotypes. This transient nature of the macrocephaly phenotype in COs may hinder the full understanding of the underlying mechanisms as well as the development of potential pharmaceutical interventions for macrocephaly.

To develop a potential pharmaceutical treatment of macrocephaly, we next conducted an evaluation of the efficacy of various small molecules inhibiting distinct signaling pathways to correct the aberrant structure of VZ and impaired early neuronal differentiation in CO^ASD^ (Figure 3). From day 7 to 15, when the typical macrocephaly phenotypes (malformation of VZ, increased proliferation, and decreased differentiation of neural progenitor cells in VZ) become evident, we treated CO^ASD^ with small molecules including DAPT, a γ‐secretase inhibitor blocking Notch signaling, SU5402, an inhibitor of FGF signaling, PD0325901, an inhibitor of the MEK/ERK pathway, and cAMP, a modulator of neural differentiation (Figure 1A). Each of these small molecules is known to regulate the proliferation and differentiation of neural progenitor cells [55]. Our investigation aimed to identify which small molecule(s) could effectively adjust the observed abnormalities in CO^ASD^, thus presenting a promising concept for pharmacological interventions in treating macrocephaly.

**FIGURE 4:**
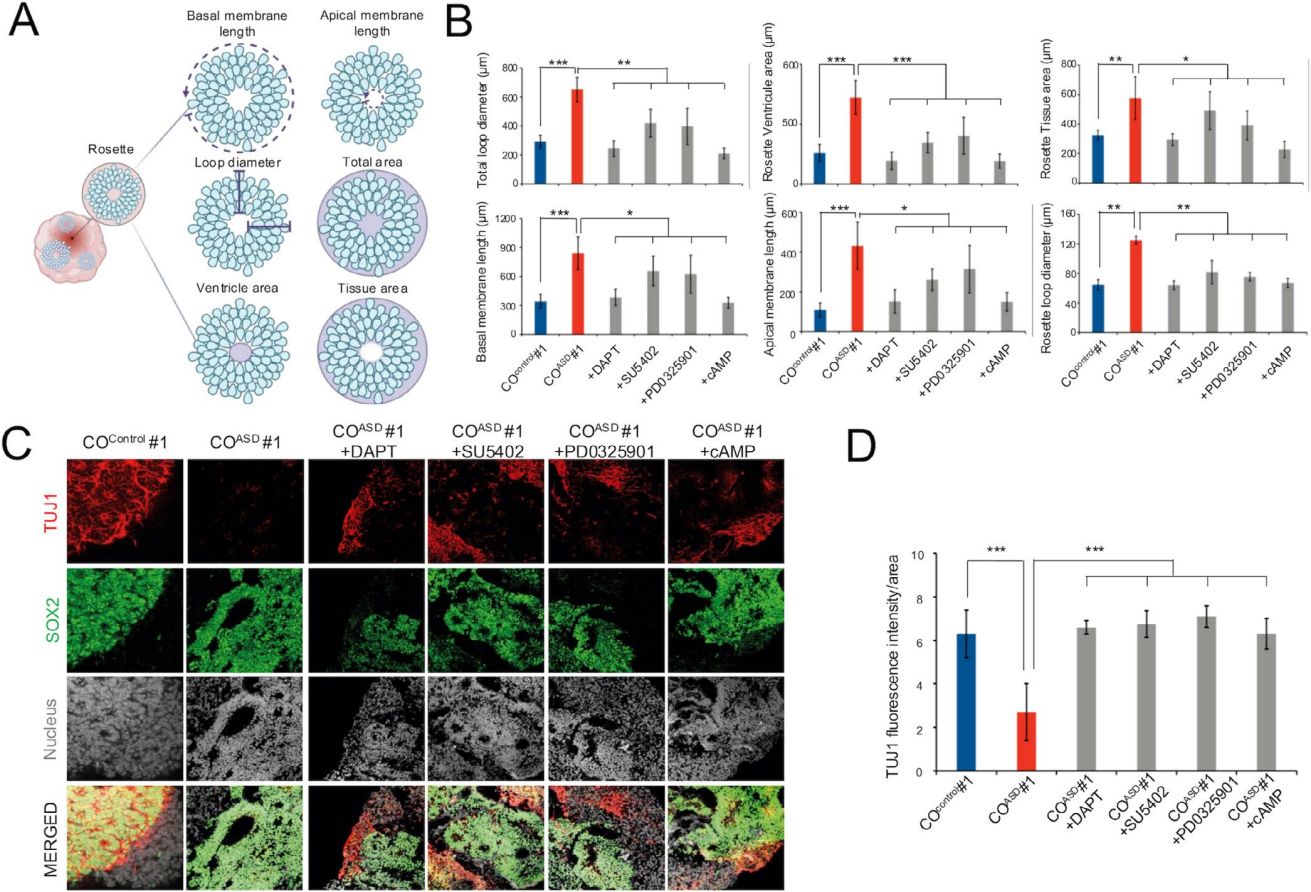
Small molecules rescue macrocephaly‐like phenotypes. (A) Illustration explaining geometric analysis measuring both apical and basal membrane lengths of the rosette, loop diameter of the rosette, total rosette area, rosette ventricle area, and rosette tissue area. (B) The effects of small‐molecule treatment were evaluated. (C) Confocal images showing expression patterns of SOX2/TUJ1 in small molecule‐treated CO^ASD^. CO^control^ and CO^ASD^ were used as controls. (D) Average fluorescence intensity of the TUJ1 signal in small molecule‐treated CO^ASD^. CO^control^ and CO^ASD^ were used as controls. Scale bars represent 100 μm. Data are presented as mean ± SD from three independent experiments. *p < 0.05, **p < 0.01, ***p < 0.001. CO^ASD^, COs from ASD hiPSC lines; CO^Control^, COs from control hiPSC lines.

To evaluate the effect of each small molecule on the tissue cytoarchitecture of COs, particularly regarding the malformation of VZ, we performed geometric analysis of COs by measuring various morphological parameters, including apical and basal membrane lengths of rosettes, loop diameter of rosettes, total rosette area, rosette ventricle area, and rosette tissue area (Figure 4A). As previously described (Figure 3A), all the geometric values were significantly higher in CO^ASD^ compared to CO^control^ (Figure 4B), contributing to the macrocephaly‐like phenotype in CO^ASD^. Remarkably, upon treatment of each small molecule (from day 7 to 15), all these values in CO^ASD^ were decreased to levels similar to those of CO^control^ (Figure 4B). Notably, both DAPT and cAMP exhibited the most dramatic rescue effects in alleviating the macrocephaly‐like phenotype of CO^ASD^, leading to the acquisition of a normal tissue architecture in CO^ASD^ (Figure 4B).

Next, we next performed immunohistochemical analysis of TUJ1 to investigate whether the small molecule treatment could also rescue the neuronal differentiation defect in CO^ASD^ (Figure 3E). Compared to the non‐treated CO^ASD^, the small molecule‐treated CO^ASD^ exhibited accelerated neural differentiation, as evidenced by TUJ1 staining (day 15) (Figure 4C). Furthermore, the small molecule treated CO^ASD^ showed a significant increase in the number of TUJ1^+^ neurons, reaching levels similar to those observed in CO^control^ (Figure 4D). In contrast, the number of Ki67^+^ proliferating cells was reduced upon treatment with each small molecule (Figure S4A,B) without causing significant cell death (Figure S4C). Among the various small molecules, DAPT treatment was the most effective (Figure 4B and S4A). Consequently, the DAPT‐treated CO^ASD^ at day 45 of differentiation showed similar numbers of both SOX2^+^ and TUJ1^+^ cells compared to CO^control^ (Figure S4D). Thus, our data indicate that the in vivo‐like pathophysiology of CO^ASD^, including macrocephaly and impaired early neuronal differentiation, could be rescued and restored to levels similar to those of CO^control^ by the treatment of small molecules, such as DAPT, resulting in the acquisition of a normal tissue architecture.

### 3.4 Differentiation Defect Into GABAergic Neurons in ASD Organoids

E/I imbalance has been highlighted as one of the major pathophysiological mechanisms underlying ASD [56]. Thus, we next tried to investigate whether this E/I imbalance could also be replicated in the patient‐derived COs. To achieve this, we generated COs using DAPT for efficient neural induction in CO^ASD^. Under this condition, both CO^control^ and CO^ASD^ displayed comparable sizes with similar numbers of TUJ1^+^ neurons (day 45) (Figure S4D).

**FIGURE 5:**
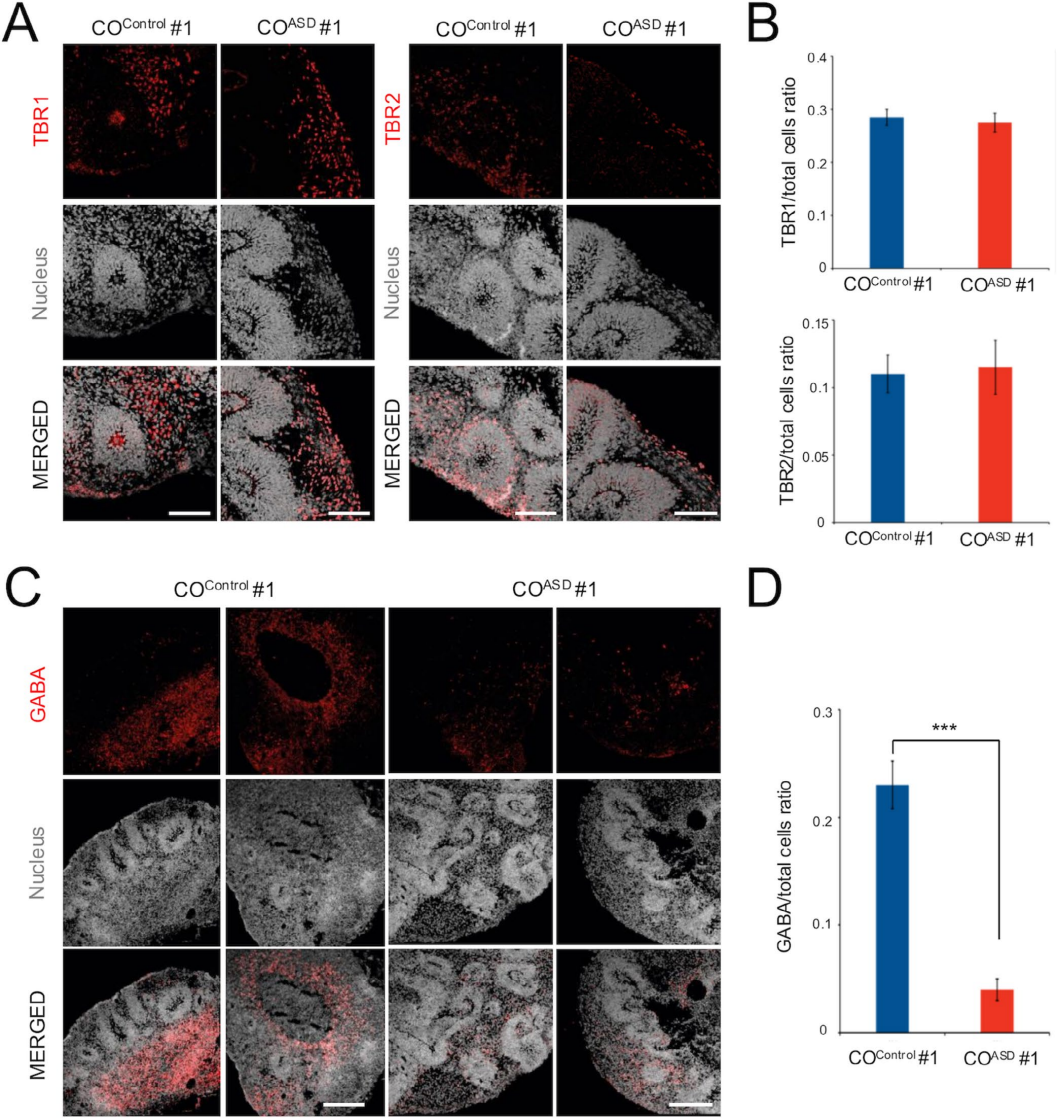
Differentiation defect into GABAergic neurons in patient‐derived COs. (A) Confocal images showing the expression pattern of TBR1 and TBR2 in CO^control^ and CO^ASD^ at day 45. (B) Average numbers of neurons expressing TBR1 or TBR2 in CO^control^ and CO^ASD^ at day 45. (C) Confocal images showing the expression pattern of GABA in CO^control^ and CO^ASD^ at day 45. (D) Average number of neurons expressing GABA in CO^control^ and CO^ASD^ at day 45 (*p* = 1.75x10^−10^). Scale bars represent 100 μm. Data are presented as mean ± SD from three independent experiments. CO^ASD^, COs from ASD hiPSC lines; CO^Control^, COs from control hiPSC lines.

We next performed immunohistochemical analyses to assess the distribution of both GABAergic and glutamatergic neurons in both CO^control^ and CO^ASD^. After the maturation of DAPT‐treated COs, we first examined the distribution of glutamatergic neurons by immunohistochemistry of both TBR1 and TBR2 (Figure 5A). The numbers of both TBR1^+^ and TBR2^+^ neurons were similar between CO^control^ and CO^ASD^ (Figure 5B), indicating that the differentiation process into glutamatergic neurons between CO^control^ and CO^ASD^ is comparable. In contrast, the differentiation patterns into GABAergic neurons were found to be substantially different. The number of GABAergic neurons expressing GABA was markedly reduced in CO^ASD^ compared to CO^control^, which had been matured during the same period (Figure 5C,D). This biased differentiation pattern with reduced numbers of GABAergic neurons was consistently observed in CO^ASD^ generated without DAPT as well (Figure S5A). This data indicate that although DAPT treatment could ameliorate the macrocephaly‐like phenotypes, DAPT is not sufficient to rescue the imbalance between glutamatergic and GABAergic neuronal differentiation, which is reproducibly observed in CO^ASD^. Additionally, this biased differentiation pattern with similarly reduced numbers of GABAergic neurons could also be observed in COs from different ASD patients, albeit with different degrees (Figure S5B). Collectively, our data demonstrate that the imbalance between glutamatergic and GABAergic neuronal differentiation, a key pathophysiological mechanism of ASD, could be reproduced at the organoid level. However, this biased differentiation pattern could not be rescued by small‐molecule treatment, which clearly exhibited therapeutic effects on both the malformation of VZ and the early neuronal differentiation defect.

### 3.5 IGF1 and Gabapentin Rescue the Differentiation Defect of GABAergic Neurons in ASD Organoids

**FIGURE 6:**
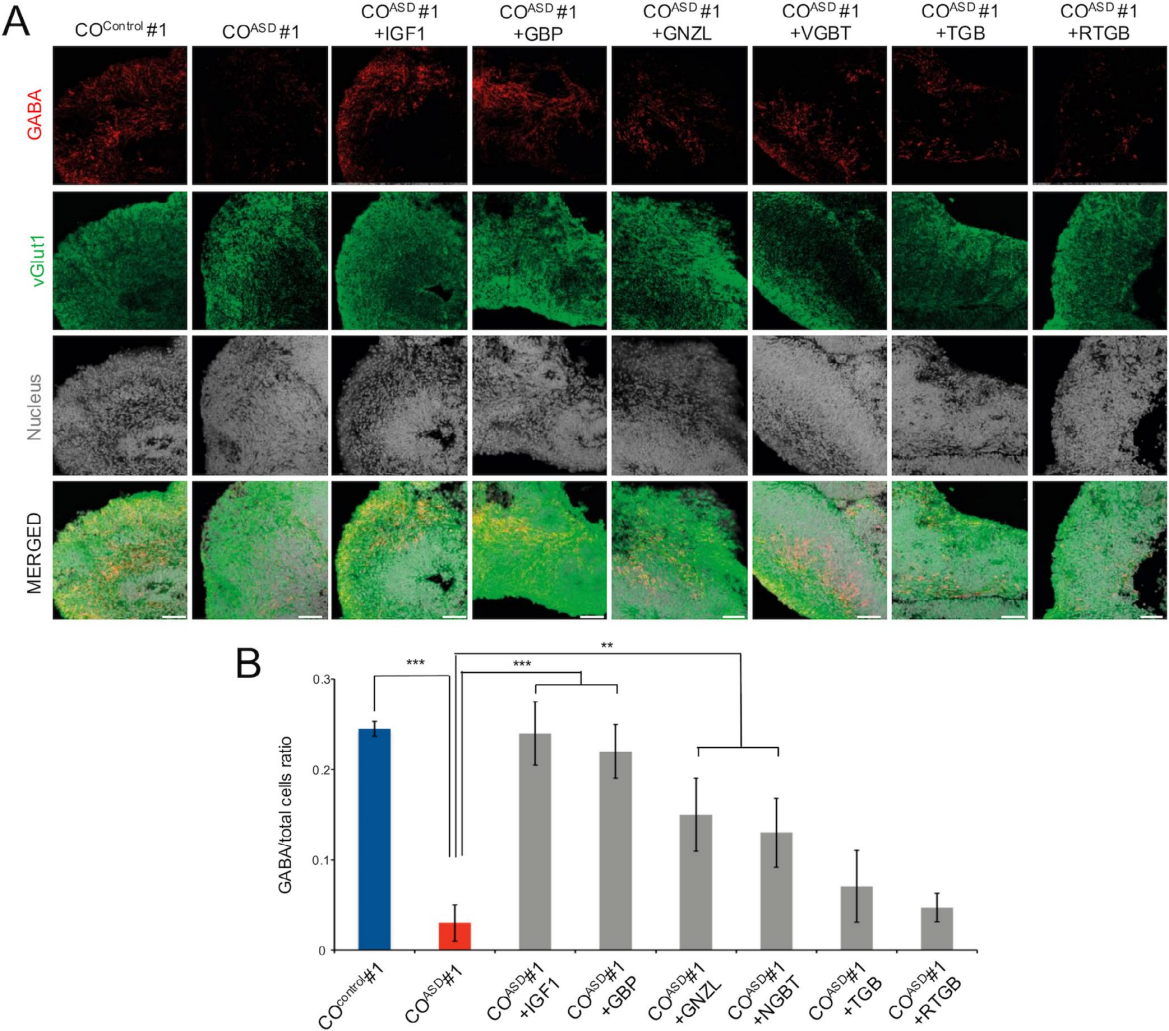
Drugs rescue the differentiation defect of GABAergic neurons in patient‐derived COs. (A) Confocal images showing GABA‐expressing neurons in drug‐treated CO^ASD^. CO^control^ and CO^ASD^ were used as controls. (B) Average number of GABA‐expressing neurons in drug‐treated CO^ASD^. CO^control^ and CO^ASD^ were used as controls. Scale bars represent 100 μm. Data are presented as mean ± SD from three independent experiments. **p < 0.01, ***p < 0.001. CO^ASD^, COs from ASD hiPSC lines; CO^Control^, COs from control hiPSC lines; GBP, Gabapentin; GNZL, Ganaxolone; RTGB, Retigabine; TGB, Tiagabine hydrochloride; VGBT, Vigabatrin.

It has been well demonstrated that the loss of CNTNAP2 function leads to ASD in humans [57] and autistic behaviors in mice [58]. In our previous study [42], we generated COs using iPSCs from Cntnap2 knockout (KO) mice. We showed that COs from Cntnap2 KO mouse iPSCs exhibited a defect in inhibitory neuron‐specific differentiation, which could be ameliorated by treating with the well‐known anti‐epileptic and anti‐convulsant drug, Retigabine [59]. In the current study, we consistently observed a defect in GABAergic neuronal differentiation in COs from multiple idiopathic ASD patients (Figure 5 and S5). Thus, we next sought to address whether we could rescue the imbalance between glutamatergic and GABAergic neuronal differentiation observed in COs by treating them with GABA agonists, including Gabapentin, Ganaxolone, Vigabatrin, Tiagabine hydrochloride, and Retigabine. Additionally, we included IGF1, which has shown its therapeutic effects for Rett syndrome [60]. It was also demonstrated that IGF1 could increase the number of GABAergic neurons during the neuronal differentiation of ASD patient‐derived iPSCs under 2D differentiation conditions [61]. For this purpose, each drug was treated on CO^ASD^ for 10 days (from day 35 to 45), and we analyzed the drug‐treated CO^ASD^ to evaluate the potential therapeutic effect of each drug on the differentiation defects of GABAergic neurons. The treatment of each individual drug did not alter the number of MAP2^+^ neurons (Figure S6), indicating that these drugs have virtually no negative effect on neurogenesis. In contrast, the numbers of GABAergic neurons were markedly increased upon treatment with each drug (Figure 6A). Among them, both IGF1 and Gabapentin exhibited the most dramatic increases in the numbers of GABAergic neurons (Figure 6A,B), indicating that the imbalance between glutamatergic and GABAergic neuronal differentiation, which was consistently observed in patient‐derived COs, could be effectively restored by treating with either IGF1 or Gabapentin. To address whether the increase in GABAergic neurons in drug‐treated CO^ASD^ could be accompanied by a reduction in glutamatergic neurons, we thoroughly examined entire sections of CO^Control^, CO^ASD^, and IGF1‐treated CO^ASD^. CO^Control^ exhibited co‐presence of both GABA‐expressing GABAergic neurons and vGlut1‐expressing glutamatergic neurons (Video S1). In contrast, CO^ASD^ showed an increased number of glutamatergic neurons but a reduced number of GABAergic neurons compared to CO^Control^ (Video S2). Surprisingly, IGF1‐treated CO^ASD^ displayed a clear reduction in glutamatergic neurons and an increase in GABAergic neurons compared to untreated CO^ASD^ (Video S3). This shift resulted in a cytoarchitecture more closely resembling that of CO^Control^, with a balanced distribution of glutamatergic and GABAergic neurons in IGF1‐treated CO^ASD^. Further studies with additional patient‐derived COs are needed to validate these findings.

All together, these results suggest that these drugs hold potential as therapeutic candidates for addressing the GABAergic neuronal differentiation defect observed in ASD, offering a promising avenue for future research and intervention strategies.

## 4 Discussion

ASD is a lifelong disorder, and the lifetime care cost for individuals affected by ASD is forecasted to be approximately 2.4 million USD [62]. The prevalence rate of ASD is steadily increasing, and the current estimated prevalence is more than 1% [63]. Despite extensive studies over the last few decades, there is still no effective treatment available for ASD. While animal model‐based studies have substantially enlarged our knowledge about the pathogenesis and pathophysiology of ASD, an increasing body of evidence clearly indicates that animal models cannot recapitulate the full spectrum of human‐specific pathophysiology of many diseases, including ASD [64]. To establish a human‐specific model for ASD, recently, iPSC technology has been employed to replicate the human‐specific pathophysiology of mostly monogenic syndromic ASD [65]. However, more than 80% of individuals affected by ASD are idiopathic [66] and thus, given the heterogeneous nature of ASD in its etiology and clinical symptoms, monogenic syndromic ASD models could not be a representative model for idiopathic ASD, which covers the majority of ASD patients. Furthermore, previous iPSC‐based approaches relying on 2D monolayer cell culture have fallen short in fully replicating bona fide disease pathophysiology observed in patient brains. Instead, they displayed the abnormal phenotypes on limited cellular levels, such as aberrances in soma sizes, neurite lengths, synaptic structures, and electrophysiological properties. Therefore, it is imperative to generate brain organoids that closely resemble the patient's brain in terms of tissue cytoarchitecture, cell type diversity, cell‐to‐cell interactions, and function. This approach would require large numbers of idiopathic ASD patients with diverse clinical symptoms to unveil the underlying mechanisms of idiopathic ASD and discover potential effective treatments tailored to this complex disorder [43].

Recent studies utilizing brain organoid technology have yielded exciting findings, showcasing the ability to phenocopy some major pathophysiological aspects of ASD in patient‐derived organoids. Notably, macrocephaly phenotypes could be observed in brain organoids derived from idiopathic ASD patients and genome‐edited iPSC lines [32, 33, 34, 35]. Additionally, phenotypes associated with the imbalance between excitatory and inhibitory neurons (E/I imbalance), a major mechanism of ASD, could also be recapitulated in brain organoids from both idiopathic ASD patients and monogenic syndromic ASD iPSC lines [36, 38, 42]. Several previous studies have consistently reported an increased number of GABAergic inhibitory neurons in patient‐derived organoids compared to healthy controls. In the current study, we were also able to observe a similar E/I imbalance‐like phenotype in idiopathic ASD patient‐derived brain organoids. However, we noted a clear difference in the detailed phenotype from previous studies. While multiple previous works described an increased number of GABAergic neurons or altered differentiation into excitatory neurons [36, 38, 42], in the current study, we observed a reduced number of GABAergic neurons in CO^ASD^ from multiple idiopathic ASD patients. This discrepancy strongly suggests that the pathophysiological phenotypes observed in idiopathic ASD patient‐derived organoids could be diverse, underscoring the need for patient‐specific ASD modeling platforms for the further translation of brain organoid technology to clinical applications, such as drug discovery. Importantly, our findings align with recent postmortem studies, which have also described a reduction of GABAergic interneurons in the prefrontal cortex of ASD patients [67, 68, 69, 70], indicating that our in vitro disease modeling data might be highly correlated with the pathological phenotypes observed in the patient brain. Furthermore, it has been noted that ASD cases with intellectual disability, rather than other comorbid symptoms, exhibited a more widespread and severe pattern of GABAergic neuronal loss [71]. Although our current study describes a significant loss of GABAergic neurons in CO^ASD^ from multiple patients, the degrees of GABAergic neuronal loss also appear to be variable among the ASD patients. Therefore, future studies aimed at deciphering the link between the altered differentiation into GABAergic neurons in CO^ASD^ and ASD pathophysiology are highly demanded to unveil the underlying mechanisms of ASD. This research may provide valuable insights into the development of targeted therapies and personalized treatments for individuals with ASD based on their specific pathological profiles reproduced in patient‐derived brain organoids.

The previous breakthrough report demonstrating the successful restoration of E/I imbalance in ASD patient‐derived brain organoids through modulating the gene expression level of FOXG1 offers an exciting concept as a potential therapeutic strategy for treating ASD [32]. However, the introduction of genetic modification in specific cell types within the complex brain tissue poses technical challenges. Although recent advances in CRISPR/Cas9 technology have demonstrated successful in vivo genome editing [72], several hurdles need to be overcome before translating in vivo genome editing technology into the clinical setting [73]. Furthermore, the potential off‐target effect of current CRISPR/Cas9 technology should also be carefully evaluated to achieve a highly reliable and safe treatment option. In contrast, a pharmaceutical approach offers the potential to accelerate the development of novel therapeutics for ASD. In the current study, we provide evidence showing the potential usefulness of ASD patient‐derived brain organoids as a personalized drug screening platform for ASD therapeutics. This approach offers a promising avenue for identifying drug candidates that can ameliorate the specific pathophysiological features of individual ASD patients.

In the current study, we found that the treatment of either IGF1 or Gabapentin rescues the imbalance between glutamatergic and GABAergic neuronal differentiation by restoring the differentiation defect in GABAergic neurons in CO^ASD^. During neural development, GABA plays a significant role in influencing the development of various neurotransmitter systems, including glutamatergic, dopaminergic, cholinergic, serotonergic, and GABAergic systems. It modulates cell survival, growth, differentiation, and synaptic maturation [74, 75, 76]. Given the crucial role of the GABAergic system in neurodevelopment, there is growing concern regarding the impact of excessive or deficient GABA levels during early development on human neurodevelopmental outcomes, as well as its potential as a therapeutic target. For example, the use of antiseizure medications, including GABAergic drugs, during pregnancy has been associated with adverse effects on neurodevelopment. However, evidence for a causal relationship is lacking for some medications, and the mechanisms of action remain poorly understood [77, 78, 79, 80]. Additionally, new targets such as KCC2, which modulates chloride concentration in developing neurons and influences GABAergic activity, have been implicated in the manifestation and symptomatic regulation of neurodevelopmental disorders [80]. Although the effects of IGF1 on GABAergic neurodevelopment are not yet fully understood, IGF1 is known to play important roles in neurodevelopment both prenatally and during the early postnatal period, as well as in the plasticity and remodeling of neural circuits throughout adolescence and adulthood [81, 82]. Recently, it has been suggested that inhibition of IGF1 signaling induces developmental defects in mice that resemble human preterm brain disorders, which can be reversed with a GABAergic modulator [83]. Moreover, IGF1 has been reported to increase GABAergic transmission in GnRH neurons by modulating endocannabinoid signaling [84]. These findings suggest that IGF1 may influence GABAergic neural development, potentially contributing to the symptomatic derangements observed in human neurological disorders, particularly neurodevelopmental disorders such as ASD and attention‐deficit/hyperactivity disorder (ADHD).

While both iPSC and organoid technologies hold great potential as advanced in vitro platforms for disease modeling and ASD drug discovery, significant variability issues must be addressed before brain organoid technology can be translated into clinical or pharmaceutical applications [85]. Beyond the inherent heterogeneity of ASD etiology and pathophysiology among individuals, several technical challenges contribute to variability. First, hiPSCs derived from ASD patients exhibit substantial variability due to differences in reprogramming methods, lab‐to‐lab technical variations, and pluripotency states. Second, hiPSC‐derived brain organoids often show considerable heterogeneity across laboratories, given the lengthy and complex differentiation protocols. Finally, drug responses can vary between organoids generated from different hiPSC clones or individual patients. These variability issues make it difficult to generalize findings from in vitro disease modeling and drug testing. Establishing a high‐throughput drug screening platform for ASD requires the production of a large number of highly uniform brain organoids with minimized batch and inter‐variation. Recently, we and others have demonstrated the generation of highly uniform brain organoids using a high‐throughput screening‐compatible platform, such as 96‐ and 384‐well plates [53]. By optimizing the differentiation protocol and incorporating high‐throughput screening technologies, we can accelerate the generation of large numbers of standardized brain organoids suitable for ASD drug testing.

## 5 Conclusions

This study demonstrates that cerebral organoids from idiopathic ASD patients can successfully recapitulate multiple ASD‐related phenotypes, such as macrocephaly and E/I imbalance‐like phenotypes. We show that both macrocephaly with impaired early neuronal differentiation and E/I imbalance‐like phenotypes can be rescued and restored to levels similar to those observed in cerebral organoids from healthy controls through treatment with small molecules or drugs. Our findings provide a valuable foundation for future in vitro disease modeling and the development of early pharmaceutical intervention strategies for ASD.

## Author Contributions

S.H. and D.‐H.W. designed the experiments. S.H., X.Y., G.H., Z.‐L.J., H.L., and C.P. performed the experiments. S.H., X.Y., G.H., Z.‐L.J., K.F., X.Y., L.W., H.Y., K.H., C.Y.S., D.‐H.W., C.H., X.J., S.Z. W.Z., N.‐H.K., K.‐P.K., L.W.Z., and D.W.H. analyzed the data. D.W.H. wrote the manuscript.

## Ethics Statement

This study was reviewed and approved by the Institutional Review Board of the Seoul National University Bundang Hospital, and all participants provided informed consent.

## Conflicts of Interest

The authors declare no conflicts of interest.

## Supporting information


Appendix S1.

**Supplementary Video 1.** Video showing the distribution of GABAergic neurons (red) and glutamatergic neurons (green) in COControl at day 60.

**Supplementary Video 2.** Video showing the distribution of GABAergic neurons (red) and glutamatergic neurons (green) in COASD at day 60.

**Supplementary Video 3.** Video showing the distribution of GABAergic neurons (red) and glutamatergic neurons (green) in IGF1‐treated COASD at day 60.

## Data Availability

The data that support the findings of this study are available from the corresponding author upon reasonable request.
