# Supplementary material for: Differentiation Defect Into GABAergic Neurons in Cerebral Organoids From Autism Patients
Source: CNS Neurosci Ther. 2025 Jun 2;31(6):e70449. doi: 10.1111/cns.70449 (PMC12129711; doi:10.1111/cns.70449)
Supplement: Supplementary file 1 — Appendix S1. [file CNS-31-e70449-s002.docx]

**Supplementary Information**

**Differentiation defect into GABAergic neurons in cerebral organoids from autism patients**

Sai Hali, Xuerui Yao, Guo Hao, Zhe-Long Jin, Kun Fu, Xiao Yun, Lin Wang, Heejeong Yoo, Hyeonwoo La, Chanhyeok Park, Kwonho Hong, Chan Young Shin, Dong-Hun Woo, Choongseong Han, Xiong Jin, Shifeng Zhu, Wenquan Zou, Nam-Hyung Kim, Kee-Pyo Kim, Leshuai W. Zhang, and Dong Wook Han

**Supplementary Figures**

Supplementary Fig. 1, Supplementary Fig. 2, Supplementary Fig. 3, Supplementary Fig. 4, Supplementary Fig. 5, Supplementary Fig. 6

**Supplementary Videos**

Supplementary Video 1, Supplementary Video 2, Supplementary Video 3

**Supplementary Table**

Supplementary Table 1

**Supplementary Figures**


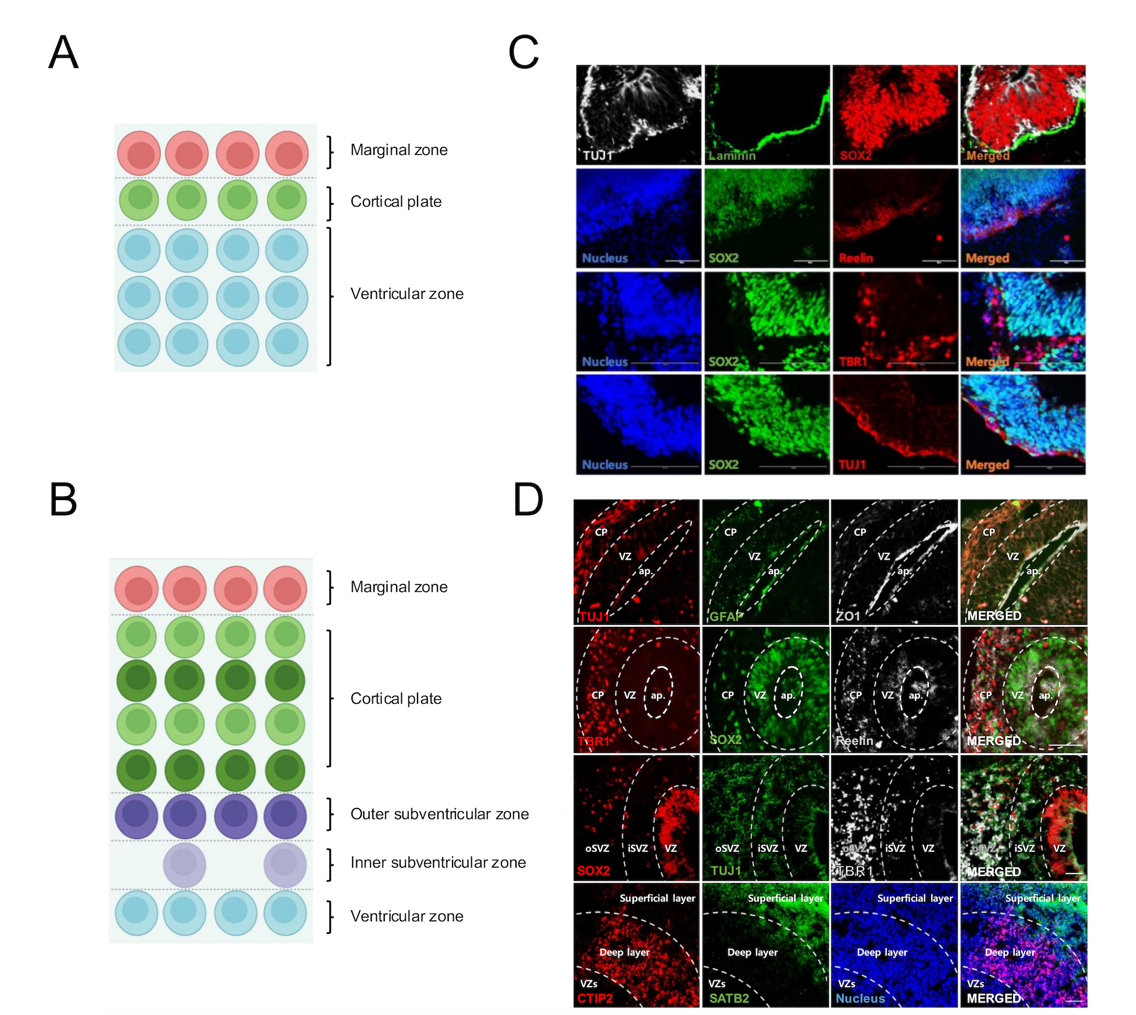


**Figure S1.** Structural analysis of early stage of COs from hPSCs. (**A**, **B**) Illustration describing the structure of developing human cerebral cortex at first (**A**) and second (**B**) trimester of gestation. (**C**) Confocal images of COs at day 22 showing SOX2+ VZ, TBR1+/TUJ1+ cortical plate, Laminin+ basement membrane, and Reelin+ marginal zone neurons. (**D**) Confocal images of COs at day 35 showing oSVZ, iSVZ, and cortical plate with CTIP2+ deep layer and SATB2+ superficial layer.

Scale bars represent 100 µm. oSVZ: outer subventricular zone. iSVZ: inner subventricular zone.


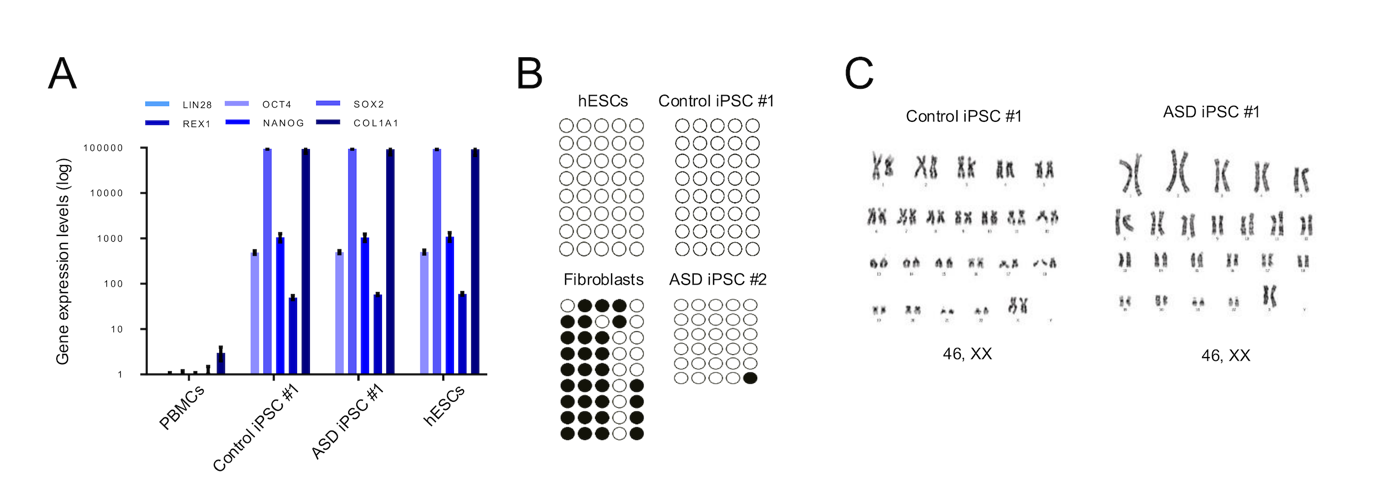


**Figure S2.** Characterization of hiPSC lines. (**A**) Expression pattern of pluripotency markers in hiPSC lines was analyzed by qPCR. All values were normalized to those of non-transduced PBMCs. Data are presented as mean ± SD of triplicate values. (**B**) DNA methylation status of OCT4 regulatory region in hiPSC lines. hESCs and fibroblasts were used as controls. (**C**) Karyotype analysis demonstrated correct chromosome content in hiPSC lines.


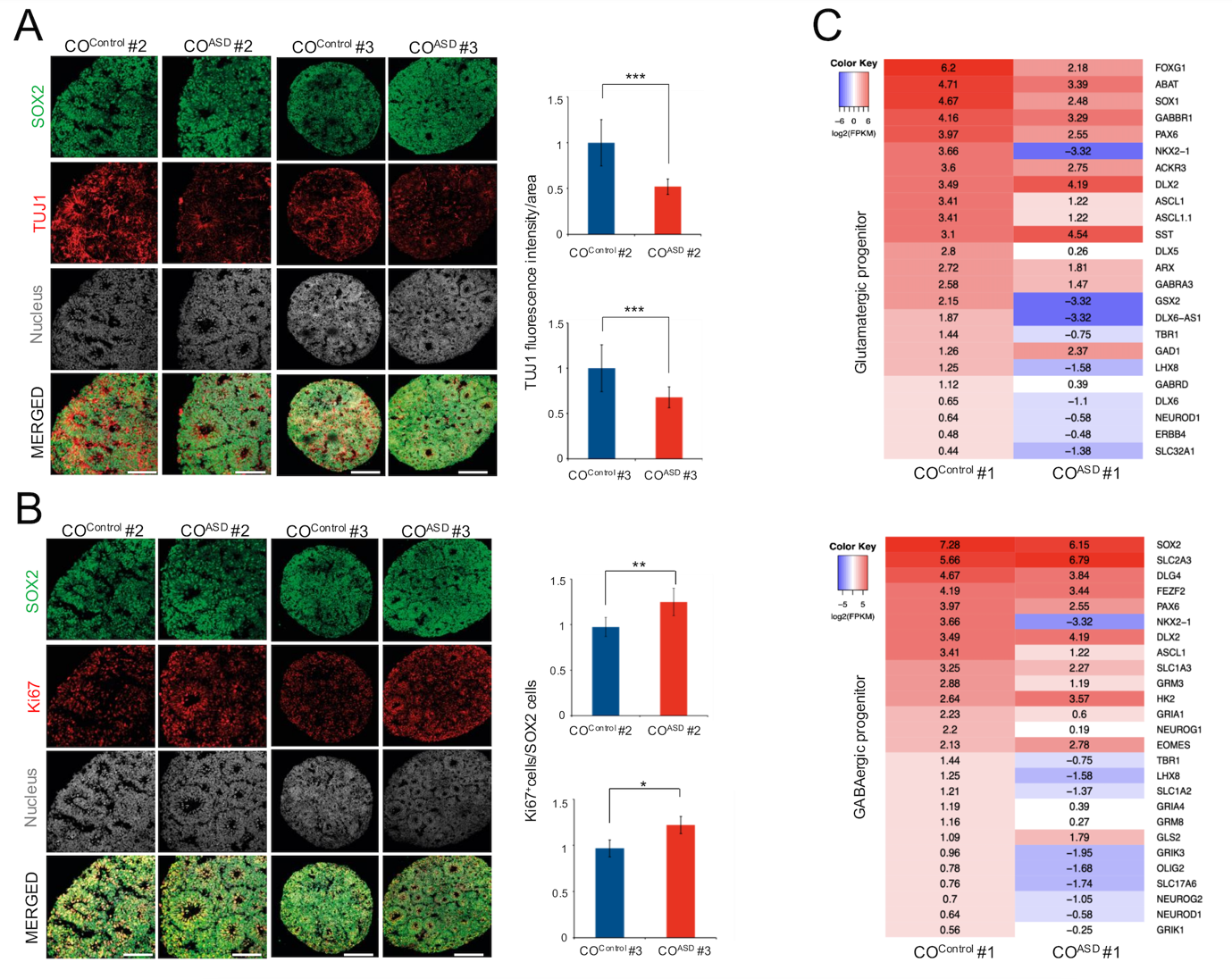


**Figure S3.** Characterization of COs from other ASD patients. (**A**, **B**) Confocal images showing TUJ1 (**A**) and Ki67 (**B**) in CO^control#2/3^ and CO^ASD#2/3^ from other ASD patients at day 15. (**C**) Heatmaps representing the comparative expression patterns of glutamatergic (upper) and GABAergic (bottom) progenitor markers between CO^control^ and CO^ASD^ at 15 days of differentiation. 15 COs per each group were subjected to RNAseq analysis. Red and blue colors represent higher and lower expression levels, respectively.

Scale bars represent 100 µm. CO^Control^: COs from control hiPSC lines, CO^ASD^: COs from ASD hiPSC lines.


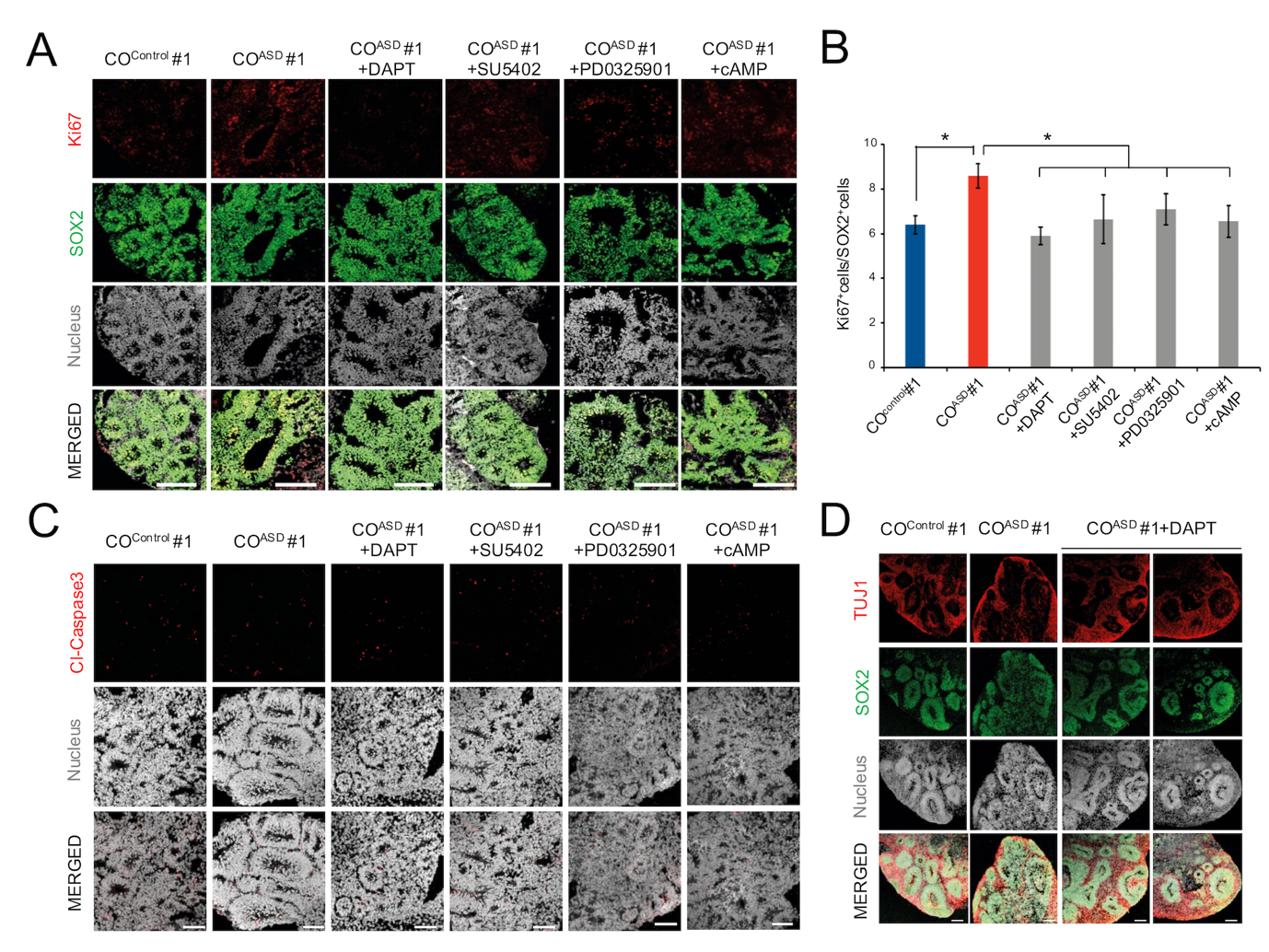


**Figure S4.** The effect of small molecules in patient-derived COs. (**A**, **B**) Confocal images (**A**) and average numbers (**B**) of Ki67+ proliferating progenitor cells in small molecule-treated CO^ASD^. CO^control^ and CO^ASD^ were used as controls. (**C**) Confocal images of cleaved-Caspase3 in small molecule-treated CO^ASD^. CO^control^ and CO^ASD^ were used as controls. (**D**) Confocal images of COs showing expression patterns of SOX2/TUJ1 in DAPT-treated CO^ASD^.

Scale bars represent 100 µm. Data are presented as mean ± SD from three independent experiments. *P<0.05. CO^Control^: COs from control hiPSC lines, CO^ASD^: COs from ASD hiPSC lines.


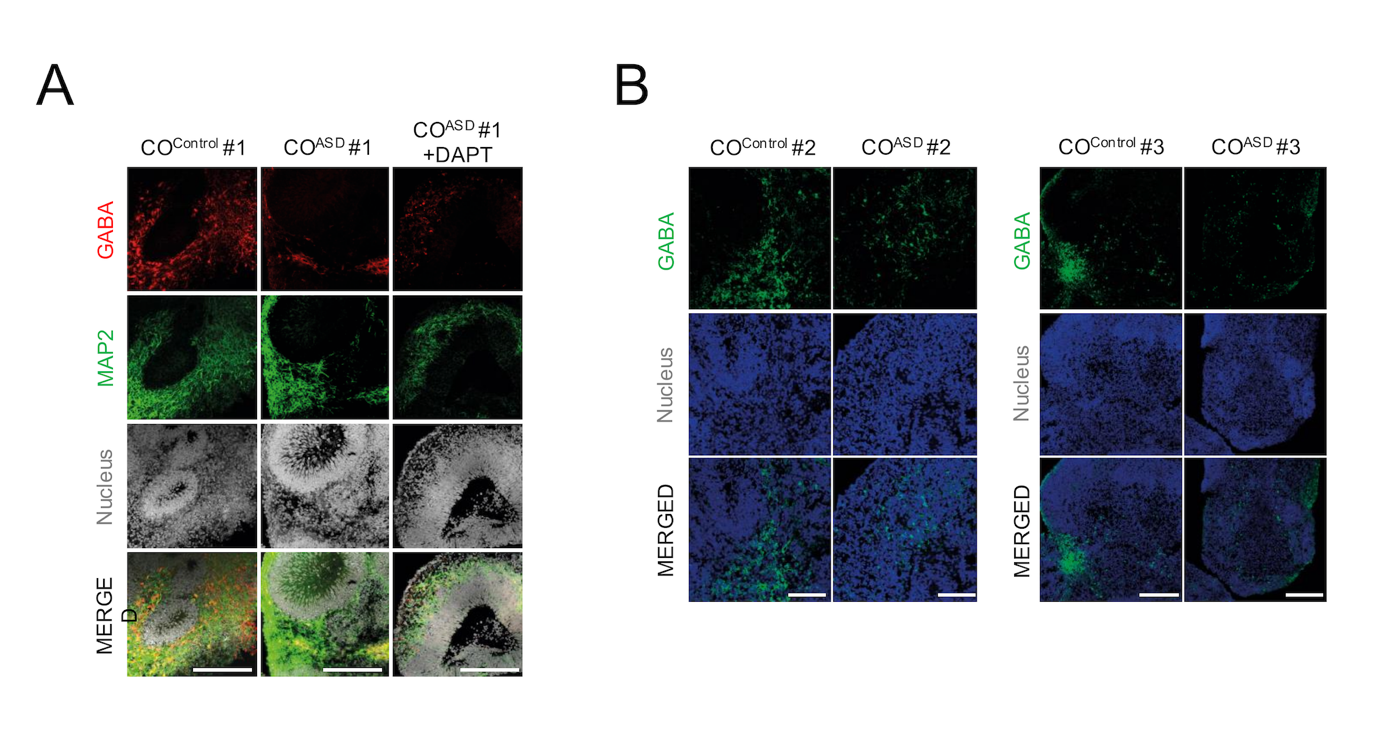


**Figure S5.** Differentiation defect into GABAergic neurons in COs from other ASD patients. (**A**) Confocal images of GABA expressing neurons in CO^control^ and DAPT-treated CO^ASD^. (**B**) Confocal images showing GABA+ cells in CO^control#2/3^ and CO^ASD#2/3^ from other ASD patients.

Scale bars represent 100 µm. CO^Control^: COs from control hiPSC lines, CO^ASD^: COs from ASD hiPSC lines.


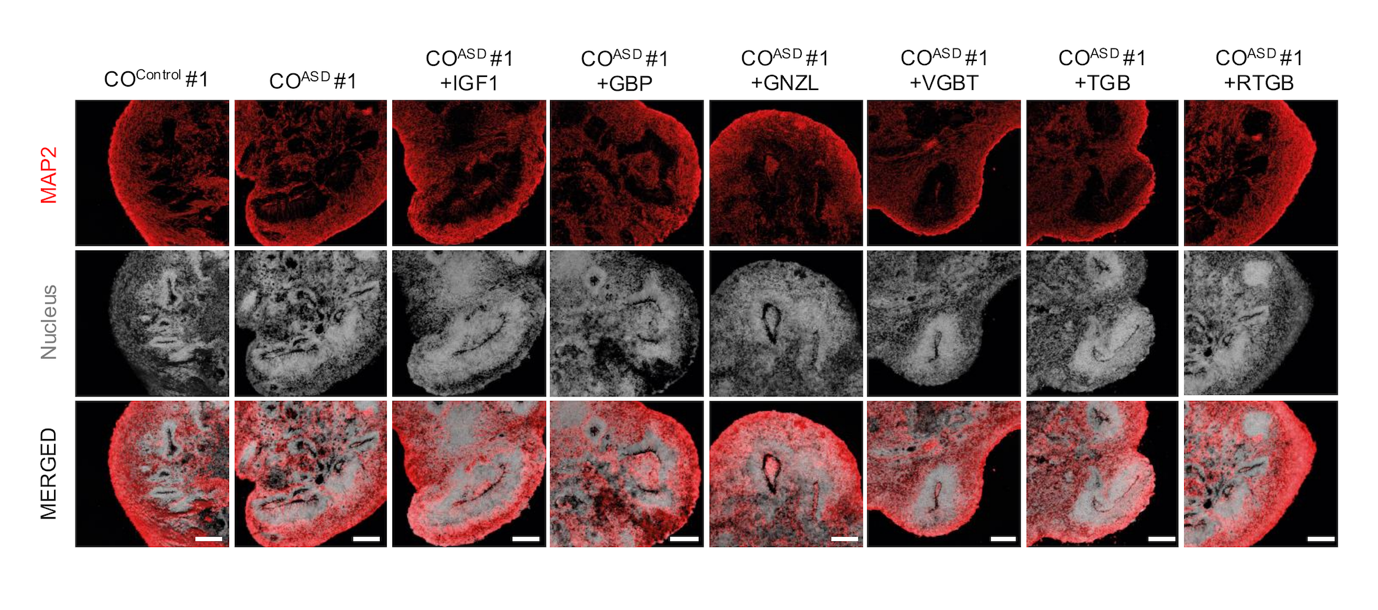


**Figure S6.** The effect of drugs on neurogenesis in COs. Confocal images of MAP2+ neuronsin drug-treated CO^ASD^. CO^control^ and CO^ASD^ were used as controls.

Scale bars represent 100 µm. CO^Control^: COs from control hiPSC lines, CO^ASD^: COs from ASD hiPSC lines, GBP: Gabapentin, GNZL: Ganaxolone,VGBT: Vigabatrin, TGB: Tiagabine hydrochloride, RTGB: Retigabine.

**Supplementary Videos**

**Supplementary Video 1.** Video showing the distribution of GABAergic neurons (red) and glutamatergic neurons (green) in CO^Control^ at day 60.

**Supplementary Video 2.** Video showing the distribution of GABAergic neurons (red) and glutamatergic neurons (green) in CO^ASD^ at day 60.

**Supplementary Video 3.** Video showing the distribution of GABAergic neurons (red) and glutamatergic neurons (green) in IGF1-treated CO^ASD^ at day 60.

**Supplementary Table**

| **Sample ID** | **Gender** | **Age at sampling** | **Proband** | **Clinical symptoms** | **Relationship with patient** |
| --- | --- | --- | --- | --- | --- |
| CO^Control#1^ | Female | 37 | No | - | Mother |
| CO^ASD#1^ | Female | 9 | Yes | Cerebral palsy |  |
| CO^Control#2^ | Female | 52 | No | - | Mother |
| CO^ASD#2^ | Female | 23 | Yes | Moderate degree without intellectual disability |  |
| CO^Control#3^ | Female | 50 | No | - | Mother |
| CO^ASD#3^ | Male | 15 | Yes | Severe degree with intellectual disability and epilepsy |  |

**Supplementary Table 1.** Summary of samples used in this study.
